# Tacrolimus‐Associated Calcium Pyrophosphate Crystal Arthritis in a Kidney Transplant Recipient: An Underrecognized Condition

**DOI:** 10.1002/ccr3.73035

**Published:** 2026-07-29

**Authors:** Matthew Wyke, Gabrielle Hitzemann, Sergio M. Maldonado‐Chaar, Veronica A. Ortigosa Serrano, Sachalee Campbell, Franco Cabeza Rivera

**Affiliations:** ^1^ Department of Internal Medicine University of Miami/Jackson Memorial Hospital Miami Florida USA; ^2^ University of Miami Miller School of Medicine Miami Florida USA; ^3^ Mount Sinai Medical Center Miami Beach Florida USA; ^4^ Division of Nephrology and Hypertension University of Miami Miller School of Medicine Miami Florida USA

**Keywords:** nephrology, pharmacology and pharmacy, rheumatology, transplantation

## Abstract

Providers who care for renal transplant recipients should be aware of the possibility of tacrolimus‐associated CPPD in patients who present with arthritis. In the case presented, a delayed diagnosis led to the progression of symptoms and avoidable disability, underscoring the need for greater clinical awareness of this condition within the transplant community.

## Graphical abstract

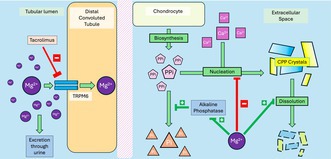

## 1 Introduction

Calcium pyrophosphate crystal deposition (CPPD) disease describes a polyarthritis resulting from abnormal CPP crystal accumulation in synovial or periarticular tissues and is associated with major disability, diagnostic uncertainty, and delays in medical care [1, 2]. Acute presentation typically includes joint pain, swelling, and erythema, and sometimes fever. Chronic hypomagnesemia is a known risk factor for CPP crystal deposition [3]. Tacrolimus has a known adverse effect of hypomagnesemia due to renal magnesium wasting [4]. There have been numerous case reports that describe CPPD flares in liver and allogeneic hematopoietic cell transplant (allo‐HCT) patients associated with tacrolimus use and the resultant hypomagnesemia caused by the drug [5, 6, 7]. Interestingly, despite the kidney being the most commonly transplanted organ, only one prior case has described tacrolimus‐associated acute monoarticular CPPD related to hypomagnesemia in a renal transplant recipient with graft failure [8]. We report a case of tacrolimus‐associated CPPD presenting as severe recurrent polyarthritis in a renal transplant recipient with preserved graft function.

## 2 Case History/Examination

A 78‐year‐old male was admitted to our institute for evaluation of bilateral lower extremity swelling for 2 weeks and right lower extremity pain and erythema for 1 day. His medical history included a living donor renal transplant (30 years prior), chronic hypomagnesemia, thyroidectomy due to thyroid carcinoma, and recently diagnosed heart failure with reduced ejection fraction. He had been on tacrolimus, mycophenolate mofetil, and steroids for immunosuppression. His chronic hypomagnesemia dated back to at least 6 years prior, and historically was not present before the transplant. He maintained a low baseline range of 1.3–1.5 mg/dL despite twice daily 800 mg magnesium oxide. He was euthyroid with levothyroxine supplementation. His tacrolimus dosing had been up‐titrated over the past 2 weeks as an outpatient and was increased to 2.5 mg twice daily at the time of admission.

Upon admission, he was treated for presumed cellulitis and heart failure exacerbation with empiric antibiotics and diuresis. Due to a recent history of melena, he was also placed on twice‐daily intravenous pantoprazole. CRP and ESR were elevated on admission at 21.0 (< 0.9) and 80, respectively. Over the following 4 days, the patient developed worsening pain, swelling, and erythema of both knees and his left wrist, left elbow, and left shoulder, raising concern for polyarthritis of an unknown etiology. He also developed fever (*T*_max_ 37.8°C), bilateral extremity tremors, and altered mental status.

## 3 Differential Diagnosis, Investigations and Treatment

Initial differential diagnosis included crystal‐induced arthritis (gout, CPPD), septic arthritis, endocarditis with immune complex arthritis, and cellulitis with reactive arthritis. Left shoulder, left wrist, and bilateral knee x‐rays showed chondrocalcinosis consistent with CPPD. Magnesium level was low at 1.3, and CRP/ESR peaked at 30/120. Transthoracic echocardiogram showed no masses or vegetations. Arthrocentesis demonstrated sterile synovial fluid with a non‐septic white cell count of 33 300 and had CPP crystals.

On admission day 5 (D5), Tacrolimus was held due to a supratherapeutic 17‐h tacrolimus trough of > 30 ng/mL, and the patient was started on 24 mg oral methylprednisolone with plans to taper. Arthritic symptoms, fever, and altered mental status resolved over 2 days.

Seven days later, he was switched to his maintenance dose of prednisone, and tacrolimus was reintroduced at a dose of 2.5 mg bid. The next day, the patient developed recurrence of altered mental status and tremor. Tacrolimus level was 6.1, and the drug was held due to a change in clinical status. He again developed right knee, left wrist, and shoulder arthritis, and fever (*T*_max_ 37.9°C). Methylprednisolone was restarted at 16 mg oral daily to taper over 14 days, and the patient had improvement in mental status and resolution of pain.

## 4 Outcome

Due to severe deconditioning and weakness, he did not meet the criteria for acute inpatient rehabilitation services. He was discharged on Tacrolimus 1 mg bid with outpatient follow‐up. Due to the impact of this flare on functional status, our patient was transferred to hospice care in the outpatient setting.

Of note, our patient had 3 previous hospitalizations within the prior year that were associated with hypomagnesemia (1.2–1.4 mg/dL) and arthritic flare; however, no diagnosis of CPP arthritis was previously made.

## 5 Discussion

To our knowledge, there exists only one previous report of tacrolimus‐associated CPPD disease occurring in a renal transplant recipient within the current literature, despite previous case reports of this phenomenon being seen in liver and allo‐HCT recipients [5, 6, 7, 8, 9].

The question is raised as to why this phenomenon within renal transplantation has not been frequently described before, and how we should manage and prevent its serious consequences.

It has been hypothesized by Cadiou et al. that the hypocalcemia associated with chronic renal disease may have a preventative effect against CPP crystal formation, in contrast to the crystal‐promoting effect of hypercalcemia in primary hyperparathyroidism [6]. We believe that further studies should be done to address this hypothesis, as it could explain a lower incidence of reported cases in renal transplantation.

Of note, our patient had normocalcemia, normal PTH level, and was 30 years out of renal transplantation with normal renal function. The only previously reported renal transplant case of tacrolimus‐associated CPPD occurred in a 30‐year‐old patient with hypomagnesemia, hypocalcemia, secondary hyperparathyroidism, and recurrent gout associated with chronic graft dysfunction (eGFR 18 mL/min) [8]. In contrast, our patient had stable renal graft function, normal calcium and parathyroid hormone levels, and chronic hypomagnesemia that developed after transplantation and persisted despite magnesium supplementation, supporting tacrolimus‐induced renal magnesium wasting as the primary contributor to CPP crystal deposition. Additionally, unlike the prior report of monoarticular disease of the knee without recurrence after magnesium supplementation, our patient developed severe recurrent polyarticular CPPD with systemic manifestations and symptom recurrence following tacrolimus rechallenge. Additionally, our patient suffered substantial functional decline associated with delayed recognition of the condition.

Nonetheless, CPPD is an underrecognized condition, and there is most likely a strong component of underdiagnosis of tacrolimus‐associated CPPD in this population [10]. A previous case series of 450 patients post‐allo‐HCT identified a 3.3% incidence (*n* = 15) of crystal arthropathy on tacrolimus suggested to be due to CPPD and this was the first publication of this phenomenon in a post‐allo‐HCT population [5].

Acute CPPD may be misdiagnosed as its symptomology mimics other more common diagnoses, such as cellulitis, as was suspected in our patient [11]. Gout is a more common and well‐described entity post‐renal transplant, with guidelines dedicated to identification and management, which can lead to clinician bias [12]. Patients presenting with arthritis may not have had synovial fluid analysis and may have had misguided but effective empiric treatment intended for gout. Furthermore, only recently has there been published diagnostic criteria for CPPD disease to improve upon underdiagnosis and the lack of observational research on the condition [13]. As efforts have been made to establish validated diagnostic criteria for CPPD disease, we believe more cases associated with tacrolimus use in renal transplant patients may begin to emerge. This prompts the question of the management of this condition in this population.

**FIGURE 1:**
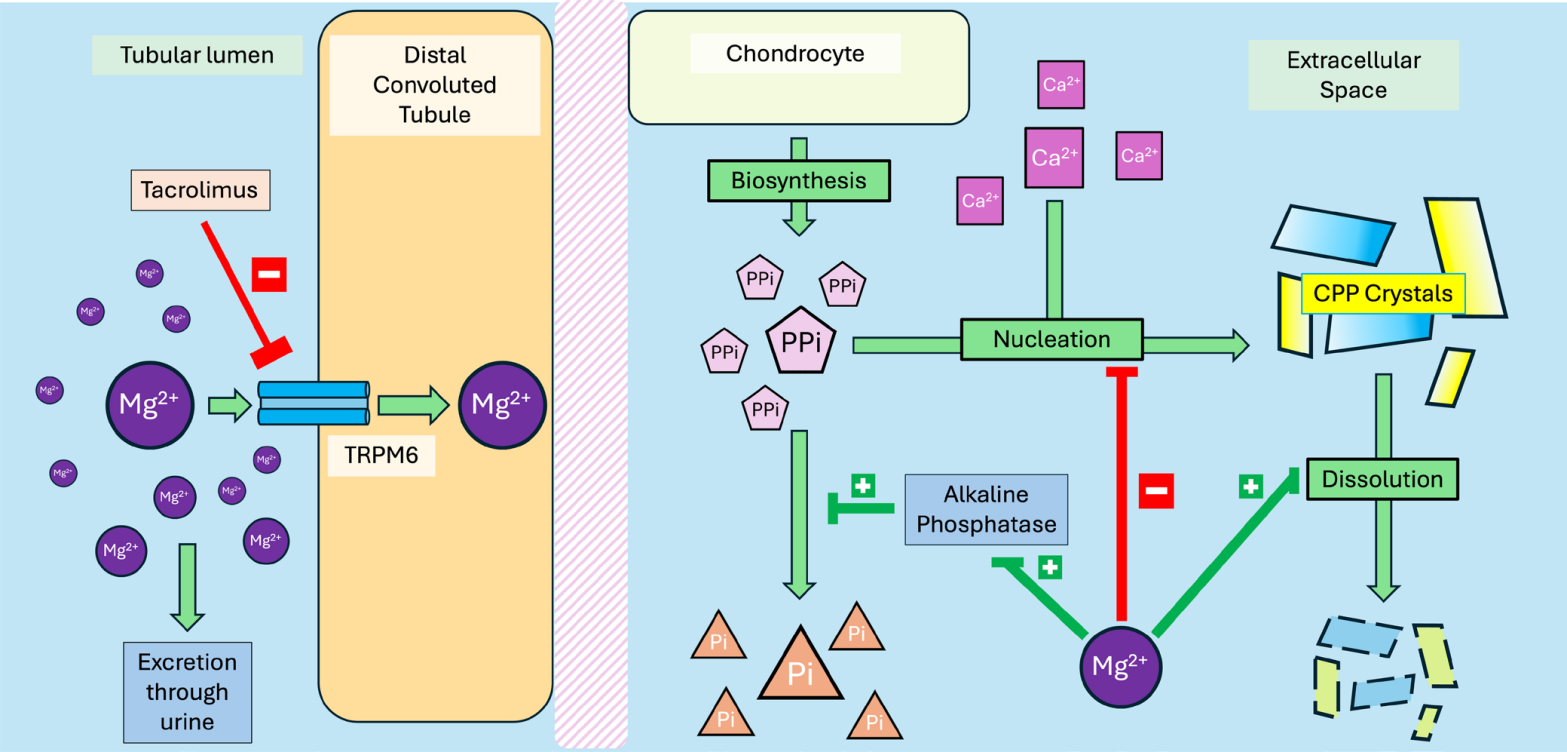
Tacrolimus induces hypomagnesemia through downregulation of apical membrane transport protein TRPM6 which is responsible for active transport of magnesium (Mg^2+^) from the distal convoluted tubular lumen.9 Hydrolysis of inorganic phosphate (PPi) to orthophosphate (Pi) occurs principally through magnesium‐dependent alkaline phosphatase (ALP). Decreased activity of ALP in hypomagnesemia thereby leads to increased PPi concentration and predisposes to CPP crystal formation. Magnesium also has an inhibitory effect on CPP crystal nucleation and growth and is involved in its dissolution [20, 21].

Tacrolimus induces hypomagnesemia through a renal loss mechanism in the distal part of the nephron [14]. Hypomagnesemia leads to elevated pyrophosphate levels and decreased solubility of pyrophosphate in the synovial fluid, increasing the risk of crystal formation and accumulation (Figure 1) [15]. Previous studies have shown a protective effect of magnesium correction on the prevention of acute and chronic CPPD disease. Magnesium replacement improved symptoms of CPPD disease at 6 months in a double‐blind, placebo‐controlled trial [16, 17]. We suggest purposeful magnesium correction with closely followed magnesium and tacrolimus levels while tacrolimus is still being used. In cases where magnesium does not normalize, additional sources of hypomagnesemia should be investigated. On his most recent hospitalization, our patient was treated with both proton pump inhibitors and loop diuretics, which decrease gastrointestinal magnesium absorption and exacerbate renal magnesium loss, respectively [18, 19]. Stopping medications that work synergistically to cause hypomagnesemia needs to be considered.

In terms of immunosuppressive treatment, we hypothesize that the benefits of continuing tacrolimus along with purposeful magnesium replacement plus or minus maintenance corticosteroid for prophylaxis may be preferred over using other immunosuppressants.

In another post‐liver transplant case, tacrolimus was switched to everolimus after persistent hypomagnesemia and acute CPP arthritis recurrence [6]. Everolimus is not commonly known to cause hypomagnesemia, and on 8 months of follow‐up with everolimus the patient did not have recurrence of CPP arthritis on levels of prednisone under 5 mg day, however magnesium levels were still low despite replacement [6].

Switching to mTor inhibitors may be associated with less severe hypomagnesemia but with a higher risk of acute rejection [22, 23]. Cyclosporine is currently out of favor in preference to tacrolimus and also causes hypomagnesemia post‐transplant [24].

Regarding treatment of acute flares, corticosteroids and colchicine are the mainstays of treatment of CPPD; but in a renal transplant population, corticosteroids may be preferable. Colchicine and tacrolimus can be a harmful combination as metabolism is via CYP3A4 for both drugs, which may increase their concentrations [25]. As supratherapeutic tacrolimus levels can be associated with renal failure, the risks of using colchicine are high post‐renal transplant; therefore, some prefer to use steroids for acute flares [6]. After renal transplant, NSAIDs are recommended to be avoided [12]. A short to extended course (1–2 weeks) of corticosteroids may be the safest option. In the Cenin et al. case series, 11 of 15 patients required systemic corticosteroids, which were tapered over a median of 6 days. 5 of these patients received corticosteroids added to or substituted for colchicine due to inadequate response. In the previously described case of renal transplant, neither colchicine nor intra‐articular joint injection was effective, and the patient's symptoms responded best to oral corticosteroid taper [8].

CPPD is a condition with considerable physical and psychological impact [2]. Our patient had a late diagnosis on his most recent hospitalization, resulting in significant disability. Providers who care for solid‐organ transplant recipients and patients on long‐term systemic tacrolimus should consider tacrolimus‐associated CPPD as a possible diagnosis in patients who present with arthritis. Further research needs to be conducted to facilitate the identification, prevention, and management of this important phenomenon.

## Author Contributions

**Matthew Wyke:** conceptualization, project administration, visualization, writing – original draft, writing – review and editing. **Gabrielle Hitzemann:** writing – original draft, writing – review and editing. **Sergio M. Maldonado‐Chaar:** writing – original draft, writing – review and editing. **Veronica A. Ortigosa Serrano:** project administration, writing – original draft, writing – review and editing. **Sachalee Campbell:** writing – original draft, writing – review and editing. **Franco Cabeza Rivera:** conceptualization, supervision, visualization, writing – review and editing.

## Funding

The authors have nothing to report.

## Consent

Written informed consent was obtained from the patient to publish this report in accordance with the journal's patient consent policy.

## Conflicts of Interest

The authors declare no conflicts of interest.

## Data Availability

Data sharing not applicable to this article as no datasets were generated or analysed during the current study.
